# The Updated Dual Burden of Malnutrition Among Vietnamese School-Aged Children: A Nationwide Cross-Sectional Study

**DOI:** 10.3390/nu17213446

**Published:** 2025-10-31

**Authors:** Nghia Duc Nguyen, Duong Ngoc Truong, Hop Xuan Nguyen, Ngoc Hong Nguyen, Anh Viet Nguyen, Son Ngo Duong, Huong Lan Thi Nguyen, Long Hoang Nguyen

**Affiliations:** 1 Department of Human Anatomy, Hanoi Medical University, Hanoi 10000, Vietnam; 2 Mirai General Clinic, Hanoi 10000, Vietnam; dongduong_hvqy@yahoo.com; 3 Midu MenaQ7 Joint Stock Company, Hanoi 10000, Vietnam; 4 Department of Rehabilitation, Vietlife General Clinic, Hanoi 10000, Vietnam; 5 Department of Physical Education, Hanoi Medical University, Hanoi 10000, Vietnam; 6 Institute for Global Health Innovations, Duy Tan University, Da Nang 550000, Vietnam; 7 Faculty of Medicine, Duy Tan University, Da Nang 550000, Vietnam; 8 Institute for Advanced Study in Technology, Ton Duc Thang University, Ho Chi Minh City 70000, Vietnam; 9 Faculty of Pharmacy, Ton Duc Thang University, Ho Chi Minh City 70000, Vietnam

**Keywords:** malnutrition, stunting, thinness, overweight, obesity, Vietnam, school-aged children, adolescents

## Abstract

**Objective:** To assess the prevalence and associated factors of malnutrition—including stunting, thinness, overweight, and obesity—among Vietnamese children aged 6–17 years, and to identify demographic, geographic, and behavioral correlates to inform targeted nutrition interventions. **Methods:** A cross-sectional, nationally representative study was conducted from January 2024 to June 2025 using data from the MIDU Assessment Program. A multistage stratified random sampling approach recruited 43,505 children aged 6–17 years across all regions of Vietnam. Anthropometric measurements were obtained following WHO 2007 growth reference standards. Stunting was defined as height-for-age Z-score (HAZ) < −2 SD, and overweight/obesity as body mass index-for-age Z-score (BAZ) > +1 SD. Data on demographic characteristics, sleep patterns, sports participation, vitamin K_2_ use, and pubertal status were collected via structured questionnaires. Multivariable logistic regression was used to identify factors associated with stunting and overweight/obesity. **Results:** Overall, 3.9% were stunted, 5.1% were thin, 20.7% were overweight, and 11.4% were obese; 8.6% had any undernutrition and 39.5% had any form of malnutrition. Stunting was significantly associated with being male (OR = 1.37, 95% CI: 1.24–1.52), older age—particularly 14–17 years (OR = 6.56, 95% CI: 5.48–7.84)—and residing in the Northern midlands, North Central, South Central, and Central Highlands regions. In contrast, frequent sports participation (OR = 0.76, 95% CI: 0.68–0.84), daily vitamin K_2_–MK_7_ use (OR = 0.82, 95% CI: 0.72–0.93), and having reached puberty (OR = 0.26, 95% CI: 0.22–0.30) were associated with lower odds of stunting. For overweight and obesity, lower odds were found among females (OR = 0.48, 95% CI: 0.46–0.51) and older children, while higher odds occurred among those living in the Southeast (OR = 1.45, 95% CI: 1.36–1.53) and Mekong River Delta (OR = 1.35, 95% CI: 1.24–1.48) regions. Early sleep (OR = 0.91, 95% CI: 0.87–0.95) and sports participation (OR = 1.07, 95% CI: 1.02–1.11) showed modest associations, whereas vitamin K_2_ use and puberty were not significant predictors. **Conclusions:** Vietnamese school-aged children face a significant rate of malnutrition, with regional, gender, and age disparities.

## 1. Introduction

Malnutrition, encompassing both undernutrition and overnutrition, remains a major public health challenge worldwide, particularly in low- and middle-income countries experiencing rapid socioeconomic and nutritional transitions [1]. Globally, an estimated 149 million children under the age of 5 are stunted, 45 million are wasted, and 39 million are overweight or obese [1]. Although the majority of malnutrition research focuses on children under 5 years, the school-aged and adolescent population also faces substantial nutritional risks, which can have long-term consequences on growth, cognitive development, and future health outcomes [2].

Over the past three decades, Vietnam, like many countries in Southeast Asia, has experienced rapid economic growth accompanied by significant shifts in diet, lifestyle, and health behaviors. This transition has led to the coexistence of both undernutrition and overnutrition, a phenomenon increasingly recognized as the “double burden of malnutrition” [3,4]. Across the region, nations such as Thailand, Malaysia, and Indonesia are observing similar patterns—declining rates of stunting and underweight but rising prevalence of overweight and obesity, particularly in urban and higher-income populations [5,6,7]. Recent evidence indicates that Vietnamese children aged 5–19 years continue to face substantial nutritional challenges. Approximately 16.8% are stunted, and 10–20% are underweight, with these conditions disproportionately affecting children in rural, mountainous, and ethnic minority communities [8,9,10]. The General Nutrition Survey and subsequent nationwide studies have shown similar patterns. Among 3055 children aged 5–19 years, the prevalence of stunting and thinness/underweight was around 10%, while overweight and obesity reached 14.5%. Overall, 36.5% of children experienced some form of malnutrition, and 19.7% had at least one type of undernutrition, indicating the persistence of the dual burden [11]. Malnutrition was significantly associated with age, ethnicity, and socioeconomic status, with stunting more common among older children and those from lower-income households, whereas overweight and obesity were concentrated among children from wealthier families [11]. National surveillance data further confirm this trend: in 2020, 19.0% of Vietnamese children aged 5–19 years were overweight and 8.1% were obese, representing a steep rise from 8.5% and 2.5%, respectively, in 2010. The increase was especially pronounced among boys (22.4% overweight; 10.9% obese) and urban children (24.3% overweight; 9.0% obese), with annual growth rates for overweight and obesity reaching 8.4% and 12.5%, respectively, reflecting a very rapid upward trend in weight-related disorders [12]. Nutritional disparities remain strongly associated with geographic, socioeconomic, and demographic factors. Undernutrition and micronutrient deficiencies—including iron deficiency (11%), anemia (12%), and vitamin D insufficiency (up to 48%)—are more prevalent among children in rural and ethnic minority areas, while urban children exhibit higher rates of overweight and obesity [9,10]. Over 80% of children fail to meet energy requirements, and more than 90% of those over age seven have inadequate calcium intake, reflecting both dietary insufficiency and poor diet quality [10,13,14].

School-aged children and adolescents represent a critical window for nutritional intervention. Adequate nutrition during this period is essential for optimal growth, pubertal development, and the establishment of lifelong dietary and physical activity patterns [15]. Poor nutritional status in this age group can impair academic performance, reduce physical fitness, and increase susceptibility to chronic diseases later in life, including diabetes, cardiovascular disease, and osteoporosis [1]. Moreover, adolescence provides an opportunity to correct earlier growth deficits, particularly in stunted children, if appropriate dietary and health interventions are implemented [4]. Given these considerations, understanding the prevalence and associated factors of malnutrition in Vietnamese children aged 6–17 years is crucial for informing national nutrition strategies. This study aims to provide updated, nationally representative data on the nutritional status of this age group, encompassing stunting, thinness, overweight, and combined malnutrition indicators. By identifying demographic and behavioral correlates, the findings can guide targeted, school- and community-based interventions, contributing to the achievement of Vietnam’s national nutrition targets and the Sustainable Development Goals (SDGs) related to hunger, health, and well-being.

## 2. Materials and Methods

### 2.1. Study Design and Participants

This nationwide cross-sectional study was conducted from 1 January 2024 to 30 June 2025, using data from the MIDU Assessment Program, a national initiative assessing the anthropometric and nutritional status of school-aged children and adolescents in community and school settings across Vietnam.

A multistage stratified random sampling design was employed to ensure national representativeness by age group, sex, and geographic region. In the first stage, provinces were stratified according to the seven official geographic regions—Red River Delta, Northern Midlands and Mountains, North Central Coast, South Central Coast, Central Highlands, Southeast, and Mekong River Delta—and further classified by urban or rural status. In the second stage, communes were randomly selected within each stratum, proportional to the population size. In the final stage, households within each selected commune were chosen using simple random sampling, and all eligible children aged 6–17 years residing in those households were invited to participate.

Eligibility criteria included being within the target age range, having complete anthropometric measurements, and obtaining written informed consent from parents or legal guardians.

### 2.2. Data Collection and Measurement

Data were collected by trained health personnel following standardized anthropometric procedures to ensure accuracy and consistency. Height was measured to the nearest 0.1 cm using a portable stadiometer, and weight was measured to the nearest 0.1 kg using a calibrated digital scale. For each participant, body mass index-for-age Z-scores (BAZ) and height-for-age Z-scores (HAZ) were calculated based on the 2007 World Health Organization (WHO) Growth Reference for children and adolescents aged 5–19 years [16]. This reference provides internationally comparable standards for assessing both undernutrition and overnutrition in school-aged populations.

Nutritional status was classified according to WHO criteria. Stunting was defined as HAZ below −2 standard deviations (SD) from the median of the reference population, and severe stunting as HAZ below −3 SD.

Thinness was defined as BAZ below −2 SD, and severe thinness as BAZ below −3 SD. Overweight was defined as BAZ between +1 SD and +2 SD, and obesity as BAZ above +2 SD.

The composite indicators “any undernutrition” and “any malnutrition” were derived to capture combined nutritional risks: “any undernutrition” included children who were either stunted or thin, while “any malnutrition” encompassed stunting, thinness, overweight, or obesity.

For conceptual clarity, thinness (BAZ < −2 SD) was treated as the WHO-recommended equivalent of wasting (acute undernutrition) in individuals aged 5–19 years, representing low body mass relative to height or recent weight loss.

In addition to anthropometric data, information on demographic characteristics, sleep timing, physical activity, vitamin K_2_ supplementation, and pubertal status was obtained using structured questionnaires completed by parents or legal guardians. The variable “daily vitamin K_2_ use” referred specifically to the self-reported intake of vitamin K_2_ (menaquinone-7, MK-7) dietary supplements in tablet or chewable form during the three months preceding data collection. This variable was included as an exposure because of emerging evidence linking vitamin K_2_ intake with bone mineralization and skeletal growth in children and adolescents [17,18,19,20].

### 2.3. Statistical Analysis

All statistical analyses were performed using Stata version 17.0 (StataCorp, College Station, TX, USA). Descriptive statistics were used to summarize participant characteristics. Multivariable logistic regression models examined factors associated with stunting and overweight/obesity, adjusting for potential confounders. Multicollinearity among independent variables was assessed using variance inflation factors (VIFs), all of which were below 2, indicating no collinearity concerns. Model fit was evaluated using the Hosmer–Lemeshow goodness-of-fit test (*p* > 0.05 for all models) and pseudo-R^2^ values. Sensitivity analyses excluding regional sampling weights yielded similar results, confirming the robustness of the findings. Results were presented as odds ratios (ORs) with 95% confidence intervals (CIs), and a *p*-value < 0.05 was considered statistically significant.

### 2.4. Ethical Approval

The study was reviewed and approved by the Ethics Committee of the Vietnam–Korea Institute of Medicine and Pharmacy Research and Training (Approval Code: 01.21/GCN-HDDDNCYSH-VKIM). Written informed consent was obtained from the parents or legal guardians of all participants prior to data collection.

## 3. Results

Among the 43,505 children aged 6–17 years included in the study, 52.4% were male and 47.6% were female. The largest age group was 6–10 years (54.0%), followed by 11–13 years (32.9%) and 14–17 years (13.1%). More than half of the participants reported going to bed early (53.2%), while 46.8% did not. Regarding physical activity, 48.6% frequently played sports and 51.4% did not. Daily vitamin K_2_ use was reported by 23.9% of children, whereas 76.1% did not take it regularly. In terms of pubertal status, 72.1% had not yet reached puberty and 27.9% had. By region, the largest proportion of participants resided in the Red River Delta (45.5%), followed by the Southeast (17.8%), North Central Coast (11.4%), South Central Coast (6.8%), Northern midlands and mountains (6.5%), Mekong River Delta (6.0%), and Central Highlands (6.0%) (Table 1).

Table 2 indicates that overall, 3.9% were stunted and 5.1% were thin, while 20.7% were overweight and 11.4% were obese. The prevalence of concurrent stunting and overweight was 0.8%, and stunting with underweight was 1.1%. In total, 8.6% of participants experienced any form of undernutrition, and 39.5% had any type of malnutrition.

**Figure 1:**
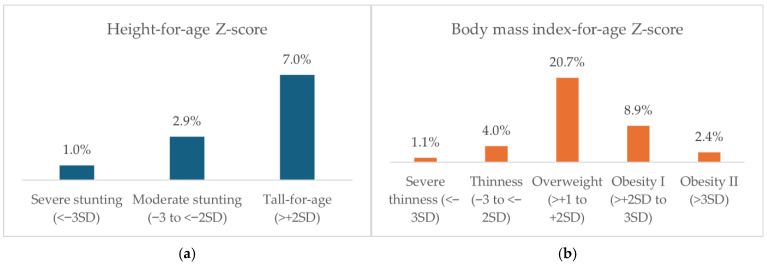
Prevalence of different HAZ and BAZ groups among children aged 6–17 years (*n* = 43,505).

Figure 1 shows the distribution of height-for-age (HAZ) and body-mass-index-for-age (BAZ) Z-score categories among 43,505 Vietnamese children aged 6–17 years. For HAZ, 1.0% of children had severe stunting, 2.9% had moderate stunting, and 7.0% were tall-for-age. For BAZ, 1.1% had severe thinness, 4.0% had thinness, 20.7% were overweight, 8.9% had obesity I, and 2.4% had obesity II.

**Figure 2:**
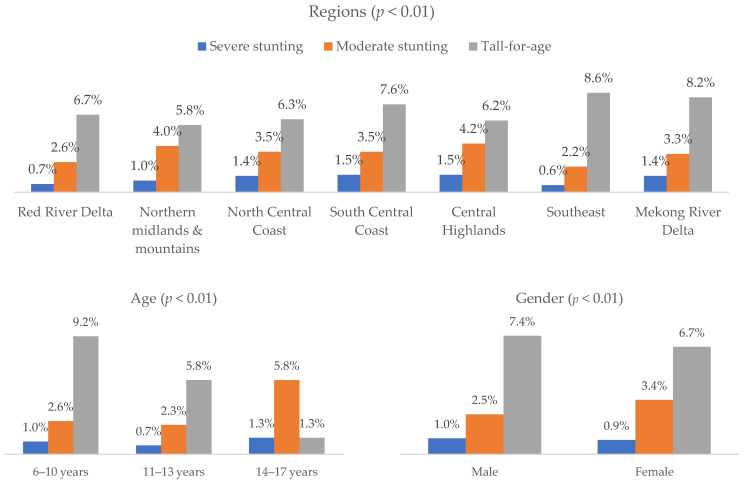

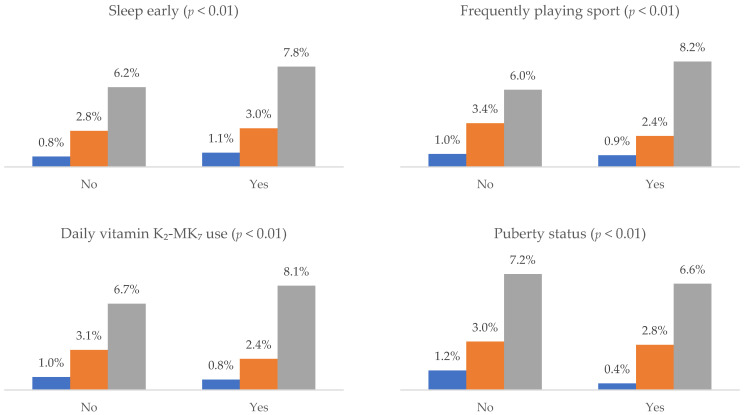
Prevalence of different HAZ groups by demographic and behavioral factors (*n* = 43,505).

Figure 2 shows that severe stunting ranged 0.7–1.3% across age groups, with tall-for-age highest at 9.2% in 6–10 years and lowest at 1.3% in 14–17 years (*p* < 0.01). Males and females had similar normal height proportions (89.1%), with tall-for-age slightly higher in males (7.4%) (*p* < 0.01). By region, severe stunting was lowest in the Southeast (0.6%) and highest in the South Central Coast and Central Highlands (1.5%), while tall-for-age ranged 5.8–8.6% (*p* < 0.01). Sleeping early, frequent sports, and daily vitamin K_2_ use were associated with slightly higher tall-for-age and lower stunting (all *p* < 0.01). Severe stunting was higher in children not yet in puberty (1.2%) than in those already in puberty (0.4%) (*p* < 0.01) (see in Table S1).

**Figure 3:**
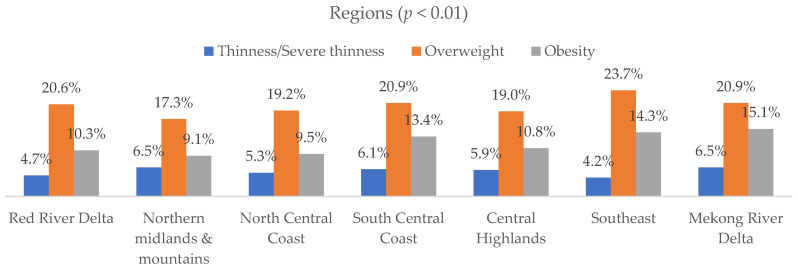

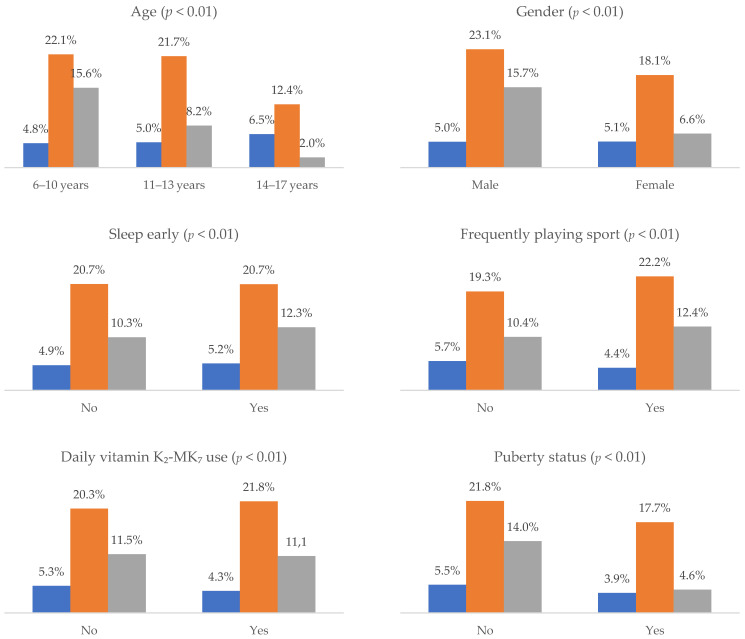
Prevalence of different BAZ groups by demographic and behavioral factors (*n* = 43,505).

Figure 3 indicated that differences across subgroups were statistically significant (*p* < 0.001). Younger children (6–10 years) showed a higher proportion of overweight and obesity, while older adolescents (14–17 years) had a greater proportion of thinness and normal BMI. Boys exhibited higher rates of overweight and obesity compared to girls. Regional variations were evident, with the Southeast and Mekong River Delta showing higher proportions of overweight and obesity. Behavioral factors such as early sleep, frequent sports participation, daily vitamin K_2_–MK_7_ use, and puberty status were also associated with variation in BMI categories (see in Table S2). 

Table 3 presents the results of multivariable logistic regression models examining factors associated with **stunting** and **overweight/obesity**. For stunting, higher odds of stunting were observed among boys (OR = 1.37, 95% CI: 1.24–1.52), older age groups—particularly those aged 14–17 years (OR = 6.56, 95% CI: 5.48–7.84)—and children from the Northern midlands, North Central, South Central, and Central Highlands regions. In contrast, children who frequently played sports (OR = 0.76, 95% CI: 0.68–0.84), used vitamin K_2_–MK_7_ daily (OR = 0.82, 95% CI: 0.72–0.93), or had reached puberty (OR = 0.26, 95% CI: 0.22–0.30) had significantly lower odds of stunting.

For overweight and obesity, lower odds were found among females (OR = 0.48, 95% CI: 0.46–0.51) and older age groups (11–13 years: OR = 0.67; 14–17 years: OR = 0.24). In contrast, children residing in the Southeast (OR = 1.45, 95% CI: 1.36–1.53) and Mekong River Delta (OR = 1.35, 95% CI: 1.24–1.48) regions had significantly higher odds. Early sleep (OR = 0.91, 95% CI: 0.87–0.95) and regular sports participation (OR = 1.07, 95% CI: 1.02–1.11) showed modest associations, while vitamin K_2_–MK_7_ use (OR = 1.05, 95% CI: 1.00–1.10) and puberty status (OR = 1.03, 95% CI: 0.96–1.10) were not significantly related to overweight or obesity.

## 4. Discussion

### 4.1. Summary of Main Findings

This study among 43,505 Vietnamese children aged 6–17 years revealed a dual burden of malnutrition, with both undernutrition (stunting, thinness) and overnutrition (overweight/obesity) present. The analysis showed notable differences in nutritional status across demographic, geographic, and behavioral groups, with distinct patterns for stunting and overweight/obesity in relation to age, sex, region, and lifestyle factors.

### 4.2. Prevalence of Malnutrition

The present study reveals a clear dual burden of malnutrition among Vietnamese children aged 6–17 years. In this study, 3.9% of participants were stunted and 5.1% were thin, whereas 20.7% were overweight and 11.4% were obese. The prevalence of concurrent stunting and overweight was 0.8%, while 1.1% exhibited both stunting and thinness. Overall, 8.6% of children experienced undernutrition and 39.5% had any form of malnutrition. These results reflect a shifting nutritional profile in Vietnamese school-aged children—from traditional undernutrition toward increasing rates of overweight and obesity—indicating an ongoing nutritional transition associated with changing diets and lifestyles [3].

Globally, the coexistence of under- and overnutrition among school-aged children has become a defining public health concern. According to Wrottesley et al. (2022) [21] and Choedon et al. (2023) [22], stunting among children aged 5–19 years ranges from 3.7% to 71.7%, while overweight and obesity can reach up to 73% in some regions. According to the World Health Organization, the prevalence of obesity among children and adolescents aged 5–19 years increased by 6.3 percentage points—from 1.9% [1.8–2.1%] in 1990 to 8.2% [7.7–8.8%] in 2022 [23], demonstrating a worsening global trend. The current Vietnamese figures are consistent with this global trend, demonstrating reduced stunting but a notable rise in excessive weight. Compared with neighboring Southeast Asian countries, Vietnam’s prevalence of overweight (20.7%) and obesity (11.4%) exceeds that of Thailand and Malaysia in similar age groups [5,6], and approaches rates reported among Indonesian urban schoolchildren [7]. This convergence suggests Vietnam is progressing along a similar nutritional trajectory, where rapid urbanization and economic growth drive shifts toward energy-dense diets and sedentary behaviors.

When compared to previous national estimates, these findings illustrate a substantial change in Vietnam’s nutritional landscape over the past decade. Earlier data from 2010 to 2020 indicated stunting at 16–17% and overweight at 8–19% among children aged 5–19 years [8,9]. The present study shows stunting has markedly declined to below 4%, while overweight and obesity have risen beyond 30% combined. This reversal aligns with the “nutrition transition” phase described by Mai et al. (2020) [24], where undernutrition continues to affect disadvantaged rural and ethnic populations, but overnutrition dominates in urban and higher-income settings. Hoang et al. (2024) also reported similar trends, noting that overweight and obesity were more prevalent in boys and children from wealthier families, while stunting persisted among those from lower socioeconomic backgrounds [12].

### 4.3. Factors Associated with Malnutrition

Malnutrition in children and adolescents is influenced by a complex interplay of biological, behavioral, and environmental factors, with gender emerging as a consistent determinant across many studies. Evidence from Vietnam shows that boys are more likely to be overweight or obese compared to girls, a pattern observed in both urban and rural settings [25,26]. For instance, in Thanhhoa City, the proportion of boys with obesity was reported to be four times higher than that of girls, indicating a marked gender disparity [26]. This gap may be partially explained by gender-specific differences in lifestyle behaviors—boys often engage in more sedentary leisure activities and have distinct dietary preferences compared to girls [27]. International literature supports these findings, suggesting that biological differences in fat distribution and hormonal influences may further contribute to this disparity [28,29,30].

Age also plays a significant role in shaping nutritional status. Overnutrition, particularly overweight and obesity, tends to be more prevalent in younger school-aged children compared to older adolescents, as shown in studies from Ho Chi Minh City [24]. Conversely, stunting is more common in younger children, particularly in the first five years of life, due to the heightened vulnerability of this period to nutritional deficits and infections [4]. While stunting reversal is possible during adolescence, the phenomenon of concurrent stunting and overweight—where children remain stunted but also gain excess weight—has been increasingly recognized, complicating both diagnosis and intervention strategies [4].

Geographic and socioeconomic factors further compound these disparities. Urban areas, such as Ho Chi Minh City and the Southeast region, report significantly higher rates of overweight and obesity compared to rural and mountainous regions [27,31]. This urban–rural gap may be attributed to differences in food environments, levels of physical activity, and household income. Regions such as the Southeast and Mekong River Delta show particularly high relative risks for child overweight or obesity compared to the Central Highlands [31]. The rapid pace of urbanization has been linked with increased access to calorie-dense foods and more sedentary lifestyles, while higher household income often correlates with greater consumption of processed and fast foods [3].

Lifestyle behaviors, including physical activity and sleep patterns, are also critical determinants of malnutrition outcomes. Physical activity not only supports healthy growth but is also associated with improved health-related quality of life in children and adolescents [32]. Meeting moderate-to-vigorous physical activity guidelines has been shown to significantly lower BMI z-scores and reduce the odds of overweight and obesity [33,34]. However, physical activity levels tend to decline during puberty, increasing obesity risk if not counterbalanced by active behaviors [35].

Sleep is another modifiable factor influencing nutritional status. Both insufficient and excessive sleep durations are linked to a higher risk of overweight and obesity in children [36,37]. Furthermore, poor sleep quality—manifested as frequent night awakenings or restlessness—has also been associated with greater obesity risk [35]. These associations highlight the need to address both the duration and quality of sleep as part of comprehensive malnutrition prevention strategies.

Biological processes such as pubertal development also intersect with nutritional status. Stunting can delay pubertal progression, resulting in shorter adult stature [38]. On the other hand, obesity—especially in girls—has been consistently linked to earlier puberty onset, a factor associated with increased risks of metabolic disorders and other health problems in adulthood [39,40,41]. The bidirectional nature of this relationship suggests that while excess adiposity can accelerate pubertal onset, early puberty does not necessarily lead to higher adiposity later in life [39].

Finally, nutrition quality, including micronutrients such as vitamin K_2_, remains essential for optimal growth. Although its role in stunting is not well studied in the included literature, vitamin K_2_ is known to support bone health and development, suggesting that adequate intake may indirectly contribute to preventing stunting in early childhood [42,43]. Taken together, these findings underscore the multifactorial nature of malnutrition, where gender, age, geography, lifestyle, biological development, and micronutrient adequacy interact to influence nutritional outcomes. Addressing these determinants in an integrated manner is essential for effective prevention and management strategies.

The findings underscore the urgent need for integrated nutrition strategies that simultaneously address the rate of undernutrition and overnutrition among children and adolescents. Such strategies should be sensitive to the regional and demographic disparities identified, recognizing that risk patterns vary by sex, age group, and geographic location. In particular, targeted interventions should be prioritized for high-risk groups, including early adolescents—where stunting and rapid weight gain often coexist—and populations in regions with elevated malnutrition prevalence, such as the Northern midlands and mountains, the Central Highlands, and the Mekong River Delta. School-based programs could incorporate tailored nutrition education, improved access to healthy meals, and structured opportunities for physical activity, while community initiatives might focus on parental engagement, dietary guidance, and affordable access to nutrient-rich foods. These interventions should also integrate behavioral components addressing modifiable risk factors, such as promoting adequate and high-quality sleep, encouraging regular physical activity, and supporting micronutrient supplementation where appropriate. By aligning public health initiatives with the local socio-cultural context, policymakers can enhance the effectiveness and sustainability of nutrition interventions.

Several limitations should be considered when interpreting these findings. First, the cross-sectional design prevents establishing temporal or causal relationships between the identified factors and nutritional outcomes; associations observed may reflect concurrent conditions rather than directional effects. Second, key behavioral variables, including sleep timing, sports participation, and vitamin use, were based on self-report, which can be affected by recall bias or social desirability bias, potentially leading to misclassification. Third, the study did not capture certain potentially important confounding variables, such as household income, parental education, dietary diversity, and food security, which are known to influence both undernutrition and overnutrition risks. Moreover, the reliance on broad regional categories may obscure intra-regional variability in food environments, health service access, and cultural practices. Lastly, while the large sample size provides strong statistical power, it also increases the likelihood that small differences reach statistical significance, which should be interpreted cautiously in terms of public health relevance. Future research should consider longitudinal designs with comprehensive dietary and socioeconomic data to better elucidate causal pathways and inform precision-targeted interventions.

## 5. Conclusions

This study reveals a notable rate of malnutrition among Vietnamese children aged 6–17 years, with both undernutrition and overnutrition present at concerning levels, varying significantly by gender, age, and geographic region. Stunting and thinness remain public health concerns, while overweight and obesity are increasingly prevalent, particularly in urban areas and among boys. The findings emphasize the need for context-specific, integrated nutrition strategies that address the full spectrum of malnutrition, incorporating school- and community-based interventions, behavioral risk factor modification, and regional targeting. Addressing these challenges holistically is essential to improving child growth, preventing long-term health consequences, and advancing national nutrition and public health goals.

## Figures and Tables

**Table 1:** Characteristics of participants.

Group	Category	Frequency (*n*)	Percent (%)
**Gender**	Male	22,775	52.4
	Female	20,730	47.6
**Age group**	6–10 years	23,481	54.0
	11–13 years	14,307	32.9
	14–17 years	5717	13.1
**Sleep early**	No	20,378	46.8
	Yes	23,127	53.2
**Frequently playing sports**	No	22,365	51.4
	Yes	21,140	48.6
**Daily vitamin K_2_–MK_7_ use**	No	33,110	76.1
	Yes	10,395	23.9
**Puberty**	Not yet	31,374	72.1
	Already	12,131	27.9
**Region**	Red River Delta	19,782	45.5
	Northern midlands & mountains	2847	6.5
	North Central Coast	4962	11.4
	South Central Coast	2968	6.8
	Central Highlands	2596	6.0
	Southeast	7741	17.8
	Mekong River Delta	2609	6.0

**Table 2:** Prevalence of nutritional and malnutrition indicators among children aged 6–17 years (*n* = 43,505).

Indicator	Frequency (*n*)	Percent (%)	95% Confidence Interval (CI)
Stunting/Severe stunting ^a^	1685	3.87	3.70–4.06
Thinness/Severe thinness ^b^	2198	5.05	4.85–5.26
Overweight ^c^	8999	20.68	20.31–21.07
Obesity ^d^	4944	11.36	11.07–11.67
Stunted and Overweight/obesity ^e^	478	1.10	1.00–1.20
Stunted and Underweight ^f^	144	0.33	0.28–0.39
Any Undernutrition ^g^	3739	8.59	8.33–8.86
Any Malnutrition ^h^	17,204	39.54	39.09–40.00

^a^ Stunting—low height-for-age (HAZ < −2 SD); ^b^ Thinness—low body mass index-for-age (BAZ < −2 SD), equivalent to wasting for individuals aged 5–19 years; ^c^ Overweight—BAZ > +1 SD to ≤+2 SD; ^d^ Obesity—BAZ > +2 SD; ^e^ Stunted and Overweight—concurrent stunting and overweight in the same individual; ^f^ Stunted and Underweight—concurrent stunting and thinness in the same individual. ^g^ Any undernutrition—defined as the presence of stunting (height-for-age Z-score [HAZ] < −2 SD) and/or thinness (body mass index-for-age Z-score [BAZ] < −2 SD); ^h^ Any malnutrition—defined as the presence of stunting, thinness, overweight, or obesity.

**Table 3:** Multivariable logistic regression for stunting and overweight/obesity among children aged 6–17 years (*n* = 43,505).

Variable	Category (Ref)	Stunting	Overweight/Obesity
		OR (95% CI)	OR (95% CI)
Sex	Male (ref: Female)	1.37 (1.24–1.52) *	0.48 (0.46–0.51) *
Age group	11–13 years (ref: 6–10 years)	1.29 (1.14–1.46) *	0.67 (0.64–0.71) *
	14–17 years (ref: 6–10 years)	6.56 (5.48–7.84) *	0.24 (0.22–0.26) *
Region	Northern midlands & mountains (ref: Red River Delta)	1.47 (1.22–1.77) *	0.80 (0.73–0.88) *
	North Central Coast (ref: Red River Delta)	1.48 (1.27–1.72) *	0.89 (0.83–0.96) *
	South Central Coast (ref: Red River Delta)	1.41 (1.17–1.70) *	1.22 (1.12–1.33) *
	Central Highlands (ref: Red River Delta)	1.60 (1.32–1.93) *	1.00 (0.91–1.10)
	Southeast (ref: Red River Delta)	0.82 (0.70–0.96) *	1.45 (1.36–1.53) *
	Mekong River Delta (ref: Red River Delta)	1.31 (1.07–1.60) *	1.35 (1.24–1.48) *
Sleep early	Yes (ref: No)	1.22 (1.10–1.35) *	0.91 (0.87–0.95) *
Frequently playing sports	Yes (ref: No)	0.76 (0.68–0.84) *	1.07 (1.02–1.11) *
Daily vitamin K_2_ use	Yes (ref: No)	0.82 (0.72–0.93) *	1.05 (1.00–1.10)
Puberty status	Already (ref: Not yet)	0.26 (0.22–0.30) *	1.03 (0.96–1.10)

* *p* < 0.05.

## Data Availability

The data presented in this study are available on request from the corresponding author due to ethical and confidentiality restrictions. The dataset contains sensitive participant information collected from surveys under the MIDU Assessment Program, and sharing is therefore limited to qualified researchers upon reasonable request and with permission from the institutional ethics committee.

## Supplementary Materials

The following supporting information can be downloaded at: https://www.mdpi.com/article/10.3390/nu17213446/s1, Table S1. Prevalence of height-for-age (HAZ) categories by demographic and behavioral factors among children aged 6–17 years (*n* = 43,505); Table S2. Prevalence of BAZ categories by demographic and behavioral factors among children aged 6–17 years (*n* = 43,505).
