# Supplementary material for: The Updated Dual Burden of Malnutrition Among Vietnamese School-Aged Children: A Nationwide Cross-Sectional Study
Source: Nutrients. 2025 Oct 31;17(21):3446. doi: 10.3390/nu17213446 (PMC12609402; doi:10.3390/nu17213446)
Supplement: Supplementary file 1 [file nutrients-17-03446-s001.zip › nutrients-3911914-supplementary.pdf]

Table S1. Prevalence of height-for-age (HAZ) categories by demographic and behavioral factors among children aged 6–17 years (n = 43,505)

Factor	Category	Severe stunting (%)	Moderate stunting (%)	Normal height (%)	Tall-for-age (%)	χ^2 (df)	p-value
**Age group**	6–10 years	1.01	2.58	87.20	9.21	685.35 (6)	<0.001
	11–13 years	0.73	2.33	91.17	5.77		
	14–17 years	1.26	5.81	91.67	1.26		
**Gender**	Male	1.03	2.52	89.07	7.38	38.14 (3)	<0.001
	Female	0.86	3.37	89.12	6.65		
**Region**	Red River Delta	0.74	2.62	89.97	6.67	159.05 (18)	<0.001
	Northern midlands & mountains	1.02	3.97	89.18	5.83		
	North Central Coast	1.43	3.47	88.81	6.29		
	South Central Coast	1.48	3.54	87.40	7.58		
	Central Highlands	1.50	4.16	88.14	6.20		
	Southeast	0.62	2.20	88.62	8.56		
	Mekong River Delta	1.38	3.26	87.20	8.16		
**Sleep early**	No	0.80	2.79	90.22	6.18	55.59 (3)	<0.001
	Yes	1.08	3.04	88.10	7.78		
**Frequent sports**	No	0.97	3.42	89.66	5.95	116.38 (3)	<0.001
	Yes	0.93	2.40	88.50	8.17		
**Daily vitamin K_2 use**	No	0.99	3.10	89.21	6.70	38.35 (3)	<0.001
	Yes	0.83	2.36	88.74	8.07		
**Puberty status**	Not yet	1.17	2.98	88.63	7.22	67.15 (3)	<0.001
	Already	0.38	2.77	90.30	6.55		

Table S2. Prevalence of BAZ categories by demographic and behavioral factors among children aged 6–17 years (n = 43,505)

Factor	Category	Thinness/ Severe thinness (%)	Normal (%)	Overweight (%)	Obesity (%)	χ^2 (df)	p-value
**Age group**	6–10 years	4.76	57.59	22.08	15.58	1500.0 (6)	<0.001
	11–13 years	4.95	65.13	21.73	8.19		
	14–17 years	6.52	79.13	12.35	1.99		
**Gender**	Male	5.04	56.21	23.08	15.68	1300.0 (3)	<0.001
	Female	5.07	70.25	18.06	6.63		
**Region**	Red River Delta	4.67	64.48	20.55	10.30	333.61 (18)	<0.001
	Northern midlands & mountains	6.50	67.12	17.32	9.06		
	North Central Coast	5.26	66.12	19.17	9.45		
	South Central Coast	6.13	59.60	20.86	13.41		
	Central Highlands	5.89	64.29	18.99	10.82		
	Southeast	4.19	57.83	23.67	14.31		
	Mekong River Delta	6.48	57.53	20.93	15.06		
**Sleep early**	No	4.88	64.11	20.69	10.32	47.67 (3)	<0.001
	Yes	5.21	61.83	20.68	12.28		
**Frequent sports**	No	5.69	64.65	19.27	10.39	142.49 (3)	<0.001
	Yes	4.38	61.04	22.18	12.40		
**Daily vitamin K_2 use**	No	5.29	62.91	20.34	11.45	24.36 (3)	<0.001
	Yes	4.29	62.85	21.77	11.09		
**Puberty status**	Not yet	5.50	58.66	21.84	14.00	1100.0 (3)	<0.001
	Already	3.90	73.86	17.69	4.55		
